# Hepatoid Adenocarcinoma of the Lung: A Case Report on a Rare Medical Condition

**DOI:** 10.7759/cureus.102211

**Published:** 2026-01-24

**Authors:** Fawad Talat, Abdul Subhan Talpur, Behrawar Ahmad, Marwa Mahjoub, Fnu Raheela, Adarsh Srinivas Ramesh, Madhuri Yalamanchili

**Affiliations:** 1 Internal Medicine, United Health Services (UHS) Wilson Medical Center, Johnson City, USA; 2 Internal Medicine, University of Toledo Health, Toledo, USA; 3 Medical Oncology, Broome Oncology, Johnson City, USA

**Keywords:** hepatoid adenocarcinoma, hepatoid adenocarcinoma of the lung, oncology, primary lung malignancy, pulmonology

## Abstract

We describe a case of an 82-year-old man who initially presented with back pain in the thoracic region and, on imaging, was found to have a right lower lobe lung nodule and a soft tissue mass eroding the posterior aspect of the right-sided ninth rib. Biopsy of this soft-tissue mass initially raised concern for metastatic HCC, but the patient was later found to have hepatoid adenocarcinoma of the lung. The patient was started on immunotherapy and a chemotherapy regimen, but unfortunately died within six months of diagnosis, highlighting the aggressive nature and course of the disease.

## Introduction

Hepatoid adenocarcinoma (HAC) of the lung is an extremely rare and aggressive, alpha-fetoprotein-producing lung carcinoma with morphological and pathological characteristics resembling hepatocellular carcinoma (HCC) and clinical features resembling lung adenocarcinoma [1]. With the first case reported in 1970, HAC is an aggressive cancer and continues to have a worse prognosis [2]. It differs from common adenocarcinoma in terms of its biological aggressiveness, poor prognosis, and clinicopathological features [2]. One of the main reasons for a worse prognosis is the lack of standardized guidelines for the treatment of this cancer due to limited available literature and research. The other important reason is that specific therapeutic targets for this rare cancer have not yet been identified [2,3]. It is important to note that the lung is one of the least common sites of HAC origin [3]. Here, we describe a case of this extremely rare form of cancer, highlighting the diagnostic and therapeutic challenges associated with this rare entity and adding to the current literature, where only a few cases have been reported to date.

## Case presentation

**Figure 1:**
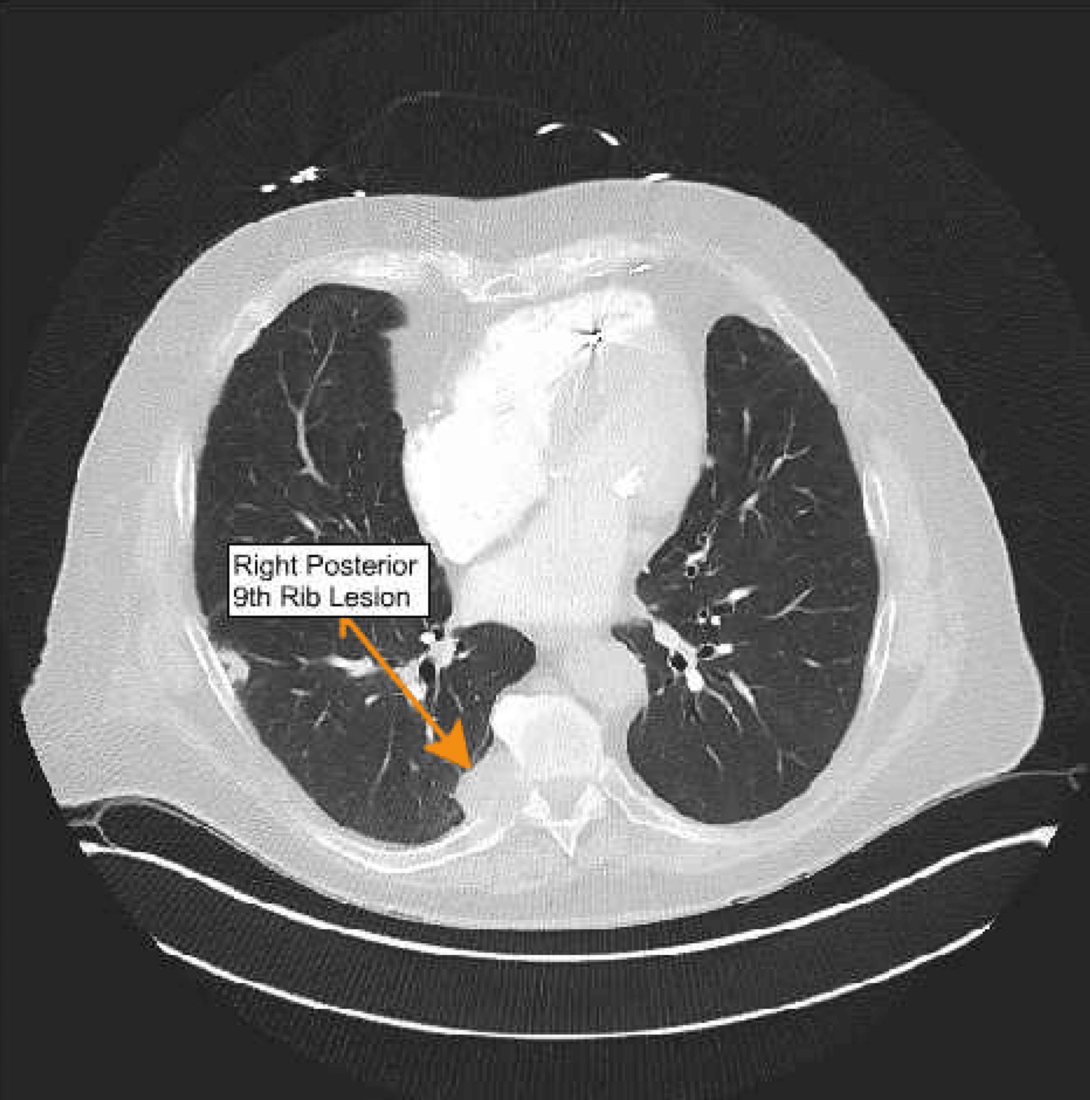
CT image showing right-sided posterior ninth rib lesion

An 82-year-old man with a past medical history of coronary artery disease status after coronary artery bypass surgery, paroxysmal atrial fibrillation, chronic kidney disease stage IIIb, essential hypertension, and hypothyroidism initially presented to the ED with severe thoracic back pain for three days. CT of the chest, abdomen, and pelvis with and without IV contrast revealed a right lower lobe lung nodule and a right lower lobe soft tissue mass eroding the posterior aspect of the ninth rib (Figure 1). The right lower lobe nodule measured 1.3 cm and was stable compared with multiple prior scans. However, the soft-tissue mass was suspicious, and tissue sampling was recommended. Of note, no abnormal mass or lesion was noted in the abdomen and pelvis. Within 10 days of the first ER visit, the patient had another ER visit for shortness of breath (SOB). A CT chest was repeated, revealing an interval increase in the size of the soft-tissue mass.

**Figure 2:**
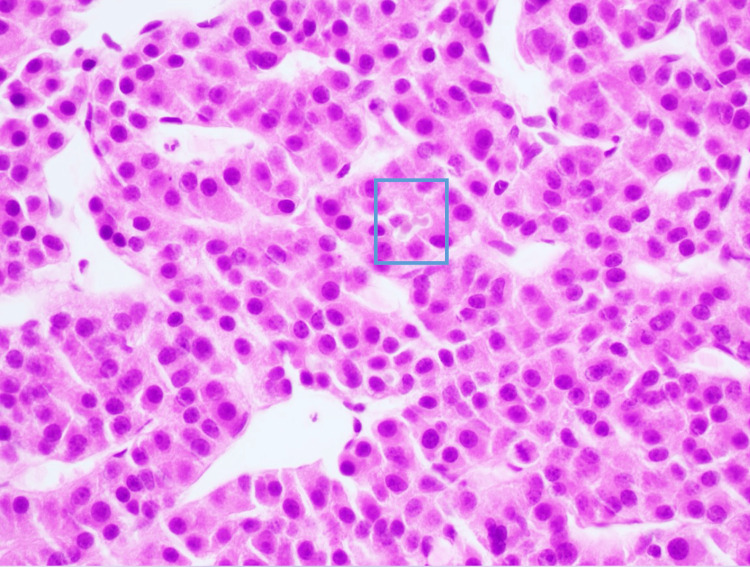
Bone biopsy showing trabecular arrangement of hepatocyte appearing cells with occasional intranuclear inclusions and bile pigment (marked with square)

Following that, the patient underwent CT-guided biopsy of the right-sided ninth rib lesion. Pathology results from the biopsied lesion revealed immunostains consistent with HCC with a high proliferative index (Figure 2). As part of further workup, the patient underwent a bone scan and a positron-emission tomography (PET) scan. The bone scan showed that the biopsied lesion did not have any abnormal uptake, and there were no other concerning findings.

**Figure 3:**
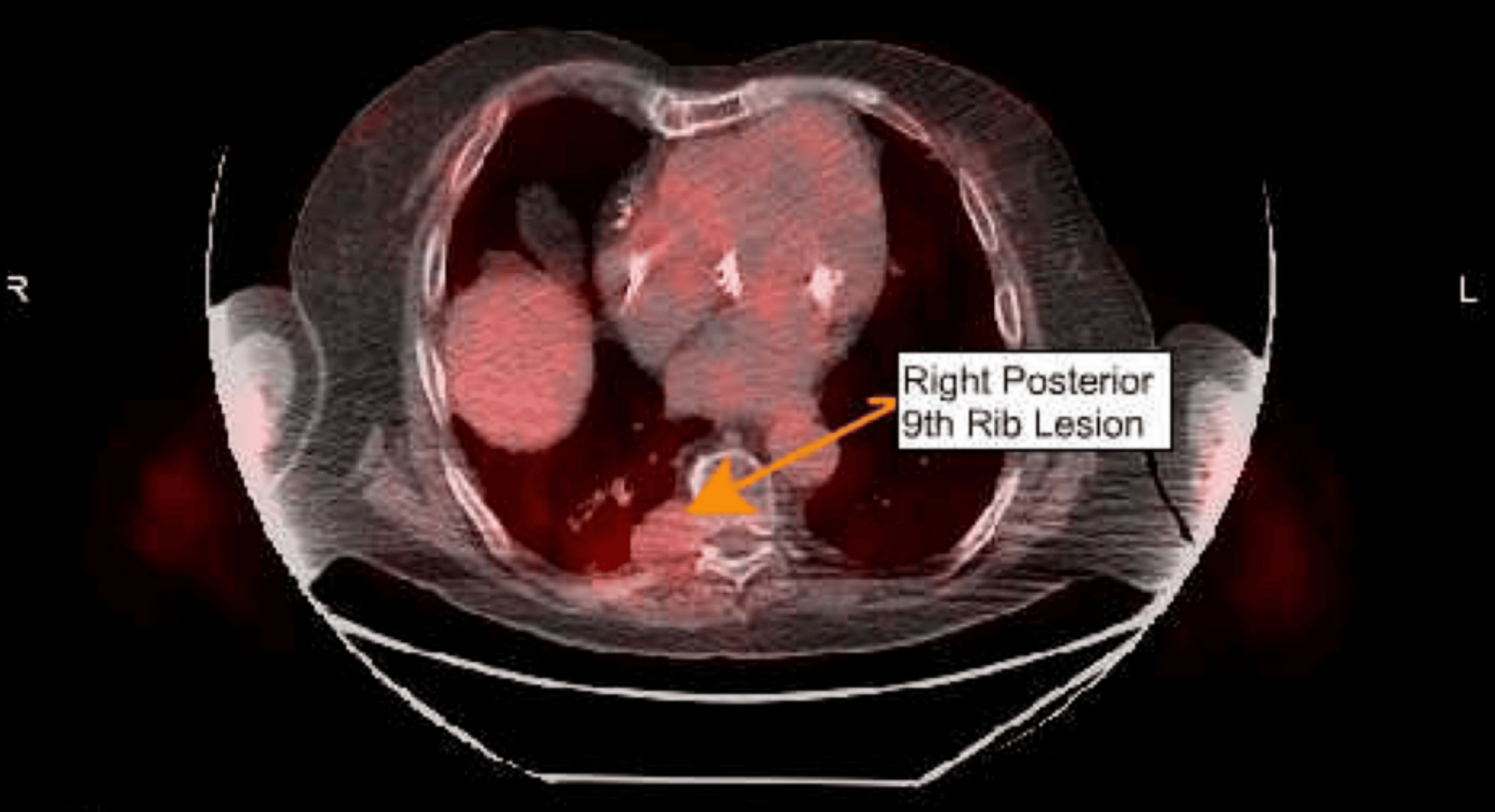
PET scan showing the hypermetabolic mass within the posteromedial aspect of the right lower lobe with extension into the posterior aspect of the ninth rib PET: positron-emission tomography

PET scan showed a hypermetabolic mass in the posteromedial aspect of the right lower lobe, extending into the posterior aspect of the ninth rib, with some interval increase in size compared with the prior study (Figure 3). The only other significant finding was hypermetabolic hilar or mediastinal lymphadenopathy concerning for metastasis, and no other suspicious liver, gastric, or other abdominal/pelvic lesions were found. For hypermetabolic mediastinal lymphadenopathy, the patient was seen by pulmonology. In addition, he underwent bronchoscopy with biopsies of the subcarinal and hilar lymph nodes. The pathology report from the biopsied lymph nodes turned out to be negative.

With a chest/lung lesion raising concern for HCC and the absence of any abnormal liver lesion, further investigation was mandated. The presence of a right lower lobe lung mass with mediastinal/hilar lymphadenopathy and the absence of any other abnormal lesion in the abdomen/pelvis raised concern for a lung origin of malignancy. In this context, a decision was made to pursue core biopsy of the lung lesion for next-generation sequencing (NGS) and tissue diagnosis. The patient ultimately underwent a core biopsy.

**Figure 4:**
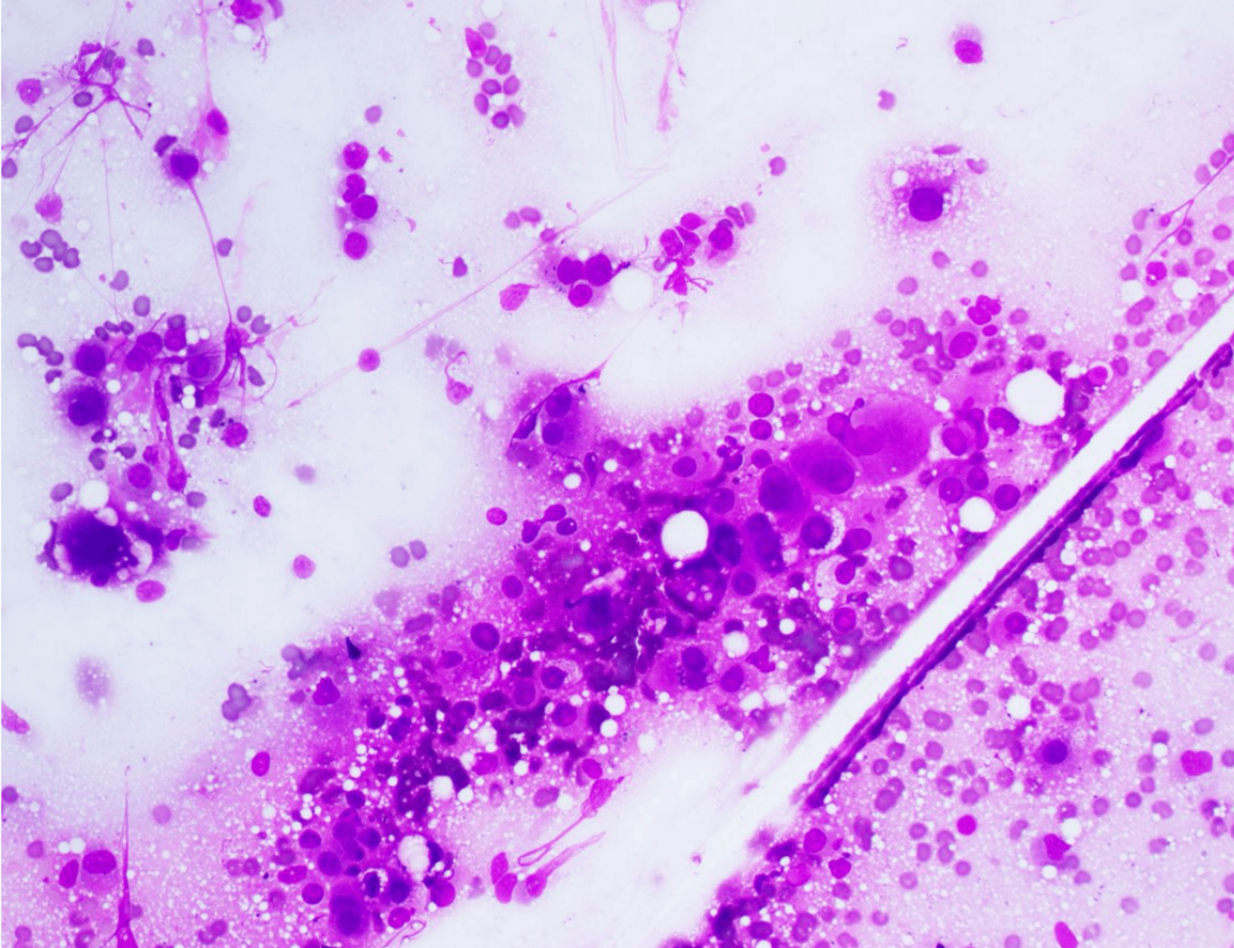
Touch imprint smear of lung biopsy, sheets of hepatocyte appearing cells with moderate cytoplasm, and occasional intracytoplasmic vacuoles

Pathology results from the core biopsy of the right lower lobe lung mass revealed hepatoid carcinoma (Figure 4). Histological findings and immunohistochemical stains supported this diagnosis. Based on PET findings and distribution of the disease process, a diagnosis of hepatoid carcinoma of the lung was made. NGS testing of the tissue revealed that the tumor was microsatellite instability stable. The tumor mutational burden was low, with no actionable mutations in the lung panel. Guardant360 (Guardant Health, Inc., Palo Alto, CA) liquid biopsy was performed, which also did not reveal any actionable mutations.

The patient was seen by two surgical oncology teams and deemed a poor candidate for any surgical intervention. After multiple opinions and consultations, a decision was made to treat the patient with chemo and immunotherapy. Given his age and frail status, he was not considered a good candidate for combination chemotherapy with immunotherapy. The patient was started on single-agent pemetrexed with pembrolizumab. Following completion of three cycles of chemotherapy, the patient was admitted to the hospital with severe SOB and was found to have grade III immune checkpoint inhibitor pneumonitis complicated by superimposed bacterial pneumonia. Unfortunately, he could not survive this hospitalization.

## Discussion

HAC is a rare entity, with histological features resembling HCC [1]. It can be found in various locations, including the stomach, pancreas, gall bladder, ovaries, uterus, and lungs [1,2]. The lung is one of the least common sites of origin for HAC, making HAC of the lung an even rarer entity [3,4]. The limited literature available overwhelmingly supports that HAC of the lungs is more common in men [3,5]. Furthermore, there is some evidence to support that cancer is more aggressive in men [3,6]. It has been suggested that estrogen may have a protective role against HAC in women, but the exact mechanism is unknown [6]. The average age of diagnosis has been described as 60 years [7].

Histologically, HAC consists of eosinophilic, polygonal cells with hyperchromatic central nuclei, arranged in a sheet-like and trabecular growth pattern, with occasional tubular regions [8]. These features make HAC virtually indistinguishable from HCC. Additionally, HAC can have components of acinar or papillary adenocarcinoma, signet-ring cells, or neuroendocrine carcinoma [6]. Differentiating between HAC and HCC is a diagnostic challenge, especially when the patient has both hepatic and liver masses. The fact that the lungs are the most common site of metastasis in HCC further complicates diagnosis [9]. Haninger et al. suggested an immunohistochemical panel, which can be used to differentiate between HAC of the lung and HCC metastatic to the lungs. This immunohistochemical panel includes a variety of cytokeratins, the monoclonal carcinoembryonic antigen, and the epithelial cell adhesion molecule (EpCAM) markers HEA125 and MOC31. HAC of the lung expresses a variety of cytokeratins (CK5/6, CK7, CK19, and CK20), EpCAM markers (HEA125 and MOC31), and napsin A. On the contrary, HCC does not express napsin A or the EpCAM markers and expresses only the cytokeratins that contribute to the intermediate filament (CK8 and CK18) [3].

In our case, lesional cells were tested for CK7, CK20, and napsin A, which turned out to be negative. However, the lesional cells were positive for hepatocyte paraffin-1 and arginase-1, which supports the identification of hepatoid differentiation [10]. Our case had a high proliferation index with Ki-67 of 40% in lesional cells. This has been reported in various other case reports and studies pertaining to HAC, pointing toward the fact that HAC should be kept in mind if histological features resemble HCC with a high proliferation index [6,8,11]. It is worth mentioning that HAC of the lung may be positive for pulmonary adenocarcinoma markers like CK7 and napsin A in some cases, but immunophenotypic expression is highly variable. Several published cases of HAC report a complete absence of CK7, CK20, and napsin A, with predominant expression of hepatocellular markers such as HepPar-1 and arginase-1 [8,11].

As of this date, no standard treatment regimen is available for patients with HAC of the lungs. This is reflected in the extremely poor prognosis associated with this cancer, a five-year survival rate of 8% [12]. The rarity of the tumor appears to be the main reason behind the lack of sufficient evidence to guide treatment strategies. In most of the reported cases, surgical intervention is employed for stage 1 and 2 patients. Deng et al., in their comprehensive review, concluded that paclitaxel plus platinum is the most commonly employed regimen and appears to be efficacious in patients with HAC. They also concluded that integrating immunotherapy appears to improve patient outcomes in lung HAC [12]. In our case, the decision to start pembrolizumab was made as no actionable genomic alteration was identified on NGS testing. There is some evidence that immune checkpoint inhibitors may benefit this rare entity [12,13].

## Conclusions

HAC of the lung is an extremely rare entity. It should be kept in the differential list especially when biopsy of lung mass shows hepatocyte appearing cells and there is no hepatic lesion or mass. Even after the diagnosis has been established, treating HAC of the lung remains a challenge. Further research is needed to elucidate optimal therapeutic strategies for this rare malignancy.

## References

[1] Nguyen P, Hui S, Robertson M (2022). Hepatoid adenocarcinoma: a wolf in hepatocellular carcinoma's clothing. JGH Open.

[2] Li M, Mei YX, Wen JH, Jiao YR, Pan QR, Kong XX, Li J (2023). Hepatoid adenocarcinoma-clinicopathological features and molecular characteristics. Cancer Lett.

[3] Haninger DM, Kloecker GH, Bousamra Ii M, Nowacki MR, Slone SP (2014). Hepatoid adenocarcinoma of the lung: report of five cases and review of the literature. Mod Pathol.

[4] Ishikura H, Kanda M, Ito M, Nosaka K, Mizuno K (1990). Hepatoid adenocarcinoma: a distinctive histological subtype of alpha-fetoprotein-producing lung carcinoma. Virchows Arch A Pathol Anat Histopathol.

[5] Bonis A, Dell'Amore A, Verzeletti V (2023). Hepatoid adenocarcinoma of the lung: a review of the most updated literature and a presentation of three cases. J Clin Med.

[6] Chen Z, Ding C, Zhang T, He Y, Jiang G (2022). Primary hepatoid adenocarcinoma of the lung: a systematic literature review. Onco Targets Ther.

[7] Cavalcante LB, Felipe-Silva A, de Campos FP, Martines JA (2013). Hepatoid adenocarcinoma of the lung. Autops Case Rep.

[8] Yang K, Jiang H, Li Q (2019). Primary pulmonary hepatoid adenocarcinoma: a case report and review of the literature. Medicine (Baltimore).

[9] Xia F, Chen Q, Li C (2025). Refining prognosis and treatment strategies beyond the Barcelona Clinic Liver Cancer stage in hepatocellular carcinoma with lung metastases: a multicenter cohort study. MedComm (2020).

[10] Mattiolo P, Scarpa A, Luchini C (2023). Hepatoid tumors of the gastrointestinal/pancreatobiliary district: morphology, immunohistochemistry, and molecular profiles. Hum Pathol.

[11] Zhuansun Y, Bian L, Zhao Z, Du Y, Chen R, Lin L, Li J (2021). Clinical characteristics of hepatoid adenocarcinoma of the lung: four case reports and literature review. Cancer Treat Res Commun.

[12] Deng H, Wang L, Li Z, Zhan T, Huang L (2024). Optimal treatment strategies for hepatoid adenocarcinoma of the lung: insights from a comprehensive analysis. BMC Cancer.

[13] Reck M, Rodríguez-Abreu D, Robinson AG (2016). Pembrolizumab versus chemotherapy for PD-L1-positive non-small-cell lung cancer. N Engl J Med.

